# HbA1c-based rather than fasting plasma glucose-based definitions of prediabetes identifies high-risk patients with angiographic coronary intermediate lesions: a prospective cohort study

**DOI:** 10.1186/s12933-023-01750-6

**Published:** 2023-03-25

**Authors:** Chenxi Song, Sheng Yuan, Kongyong Cui, Zhongxing Cai, Rui Zhang, Jining He, Zheng Qiao, Xiaohui Bian, Shaoyu Wu, Haoyu Wang, Boqun Shi, Zhangyu Lin, Rui Fu, Chunyue Wang, Qianqian Liu, Lei Jia, Qiuting Dong, Kefei Dou

**Affiliations:** 1 Cardiometabolic Medicine Center, Department of Cardiology, Fuwai Hospital, National Center for Cardiovascular Diseases, Chinese Academy of Medical Sciences and Peking Union Medical College, Beijing, China; 2 State Key Laboratory of Cardiovascular Disease, 167, Beilishi Road, Xicheng District, Beijing, 100037 China

**Keywords:** Stable coronary heart disease, Prediabetes, HbA1c, Prognosis

## Abstract

**Background:**

Prediabetes is common and associated with poor prognosis in patients with acute coronary syndrome and those undergoing revascularization. However, the impact of prediabetes on prognosis in patients with coronary intermediate lesions remains unclear. The objective of the current study is to explore the impact of prediabetes and compare the prognostic value of the different definitions of prediabetes in patients with coronary intermediate lesions.

**Methods:**

A total of 1532 patients attending Fuwai hospital (Beijing, China), with intermediate angiographic coronary lesions, not undergoing revascularization, were followed-up from 2013 to 2021. Patients were classified as normal glucose tolerance (NGT), prediabetes and diabetes according to various definitions based on HbA1c or admission fasting plasma glucose (FPG). The primary endpoint was defined as major adverse cardiovascular events (MACE), the composite endpoint of all-cause death, non-fatal myocardial infarction and repeated revascularization therapy. Multivariate cox regression model was used to explore the association between categories of abnormal glucose category and MACE risk.

**Results:**

The proportion of patients defined as prediabetes ranged from 3.92% to 47.06% depending on the definition used. A total of 197 MACE occurred during a median follow-up time of 6.1 years. Multivariate cox analysis showed that prediabetes according to the International Expert Committee (IEC) guideline (6.0 ≤ HbA1c < 6.5%) was associated with increased risk of MACE compared with NGT (hazard ratio [HR]: 1.705, 95% confidence interval [CI] 1.143–2.543) and after confounding adjustment (HR: 1.513, 95%CI 1.005–2.277). Consistently, the best cut-off point of glycated haemoglobin (HbA1c) identified based on the Youden’s index was also 6%. Restricted cubic spline analysis delineated a linear positive relationship between baseline HbA1c and MACE risk. Globally, FPG or FPG-based definition of prediabetes was not associated with patients’ outcome.

**Conclusions:**

In this cohort of patients with intermediate coronary lesions not undergoing revascularization therapy, prediabetes based on the IEC-HbA1c definition was associated with increased MACE risk compared with NGT, and may assist in identifying high-risk patients who can benefit from early lifestyle intervention.

**Supplementary Information:**

The online version contains supplementary material available at 10.1186/s12933-023-01750-6.

## Introduction

Prediabetes refers to the intermediate stage between normal glycemia and diabetes mellitus (DM), which is defined by glycemic variables that are higher than normal but lower than the thresholds for diabetes [1]. International Diabetes Federation (IDF) projections estimated that by 2045, the number of adults with prediabetes would be 548 million, corresponding to 8.4% of the world’s adult population [2]. Prediabetes is also common in patients hospitalized for coronary artery disease (CAD) without previous known diabetes mellitus history, in whom over 30% had newly detected prediabetes detected by oral glucose tolerance test (OGTT) [3, 4].

Growing evidence suggested that prediabetes was associated with poor prognosis in patients with coronary heart disease [1, 5, 6], and majority of previous studies enrolled patients with acute coronary syndrome or those who received revascularization therapy. However, the association between prediabetes and outcome in patients with coronary intermediated lesions remains unclear. In addition, there are currently five widely used definitions of prediabetes, and consensus is lacking as to the optimal definition to identify those at high risk of major adverse cardiovascular events (MACE). A better understanding of the prognostic significance of prediabetes, and which definition if any, may by most useful in the setting of coronary intermediate lesions would provide an opportunity for lifestyle modification or pharmacologic interventions to improve patients’ outcome.

The objective of the current study is therefore to examine the impact of prediabetes on outcome in patients with intermediate coronary lesions, and to compare the prognostic value of the different definitions of prediabetes.

## Methods

### Study population

Consecutive patients who underwent coronary angiography due to suspected cardiac ischemia symptoms in year 2013 were prospectively enrolled from Fuwai hospital, which locates in Beijing, China. Eligible patients had at least one lesion with angiographic stenosis of 50–70%. We excluded patients who had lesions with stenosis greater than 70%, with history of percutaneous coronary intervention (PCI) or coronary artery bypass graft surgery (CABG), underwent PCI or CABG revascularization during hospitalization, or without available data on glycemic status. The study protocol complied with the principles of the Declaration of Helsinki and was approved by the Review Board of Fuwai Hospital. Written informed consent was obtained from each participant.

### Definition of glycemic status

Patients were categorized into three groups according to prior history of diabetes, admission fasting glucose and HbA1c level. Patients were classified as diabetes mellitus, either known diabetes mellitus, defined as medical history of physician-diagnosed diabetes mellitus or taking hypoglycemic medication, or newly diagnosed diabetes, defined as the absence of known diabetes and had fasting plasma glucose (FPG) ≥ 7.0 mmol/L or HbA1c ≥ 6.5%. Prediabetes was defined as impaired fasting glucose according to World Health Organization (WHO) criteria (WHO FPG-based: 6.1−  < 7 mmol/L) [7] or the American Diabetes Association (ADA) definition (ADA FPG-based: 5.6−  < 7 mmol/L) [8], or raised HbA1c according to ADA criteria (ADA HbA1c-based: 5.7−  < 6.5%) [8] or International Expert Committee (IEC) (IEC HbA1c-based: 6.0− < 6.5%) [9]. The corresponding definition for normal glycaemia are shown in Table 1.

**Table 1:** Definitions of prediabetes according to different guidelines

	Normal glycaemia	Prediabetes
IEC HbA1c-based definition	HbA1c < 6.0%	6.0 ≤ HbA1c < 6.5%
ADA HbA1c-based definition	HbA1c < 5.7%	5.7 ≤ HbA1c < 6.5%
ADA FPG-based definition	FPG < 5.6 mmol/L	5.6 ≤ FPG < 7 mmol/L
WHO FPG-based definition	FPG < 6.1 mmol/L	6.1 ≤ FPG < 7 mmol/L

*WHO*  World Health Organization, *ADA*   american diabetes association, *FPG* fasting plasma glucose, HbA1c glycated haemoglobin, *IEC*  international expert committee

### Outcome

The primary outcome was defined as MACE, which was a composite endpoint of all-cause death, non-fatal myocardial infarction and repeated ischemia-driven revascularization. Follow-up was performed by trained cardiologists via telephone call or clinical visit at approximately 5 year post discharge. All events were carefully adjudicated by two independent clinical cardiologists, and discrepancies were dissolved by a consensus discussion with a third cardiologist. Primary outcome was defined as MACE, which was a composite endpoint of all-cause death, non-fatal myocardial infarction and revascularization.

### Laboratory analysis

Fasting blood sample was collected within 24 h on admission prior to angiography. The blood samples were collected into EDTA-anticoagulant tubes and centrifuged to obtain the plasma. Enzymatic hexokinase method was used to measure the concentrations of blood glucose. Tosoh Automated Glycohemoglobin Analyzer (HLC-723G8) was used to measure the HbA1c levels. All other laboratory measurements were performed at the biochemistry center of Fuwai Hospital by standard biochemical techniques.

### Statistical analysis

Continuous data were presented as mean ± SD or median (interquartile), and compared by using analysis of variance or the Mann–Whitney U test. Categorical variables were presented as frequency (percentage) and compared with chi-square test or Fisher’s exact test as appropriate. Restricted cubic spline was used to flexibly model and characterize the relationship between each individual glycaemic index (HbA1c and fasting glucose) and MACE, and P value for non-linearity was determined. Survival distributions were presented by Kaplan–Meier curves and compared by log-rank test. The best cutoff value in the prediction of MACE risk was defined as the cutoff point having the highest Youden index (sensitivity + specificity − 1). Univariate cox proportional hazard regression was performed to explore the association between each baseline variable and outcome, and the hazard ratio (HR) (95% confidence interval [CI]) was calculated for each variable. Multivariate cox proportional hazard regression model was used to explore the association between glycaemic status (i.e. normal glycaemia, prediabetes, DM) and outcome after the adjustment of confounding variables. Covariates are selected based on statistical and clinical significance, which included the variables with P value less than 0.05 in baseline comparison across groups and univariate analysis (Additional file 1: Table S1), as well as those clinically judged as important prognostic factors in the setting of CAD. A total of two models was used: Model 1 (the base model) adjusted for age, sex; Model 2 (fully-adjusted model) adjusted for the variables in model 1 plus medical history of hypertension, hyperlipidemia, smoking status, alcoholic consumption, body mass index (BMI), heart rate, total cholesterol, low-density lipoprotein (LDL), high-density lipoprotein (HDL), high-sensitivity C-reactive protein (hsCRP), D-Dimer and triple vessel disease. The same univariate and multivariate Cox regression analysis was performed when HbA1c and fasting glucose was modelled as a continuous variable. Spearman’s rank correlation analysis was performed to explore the association between HbA1c/fasting glucose level and hsCRP, LDL, HDL and total cholesterol. Subgroup analysis was performed to investigate whether the association between glycemic parameters and MACE differed by subgroup according to age, sex, smoking status and medical history of hypertension, and the P value for interaction test was determined. The statistical analysis was performed by SAS software Version (SAS Institute, USA) and figures were generated by GraphPad Prism version 7.0.0 for windows (GraphPad Software, San Diego, California USA).

## Results

### Categories of abnormal glucose metabolism according to different definitions

**Fig. 1:**
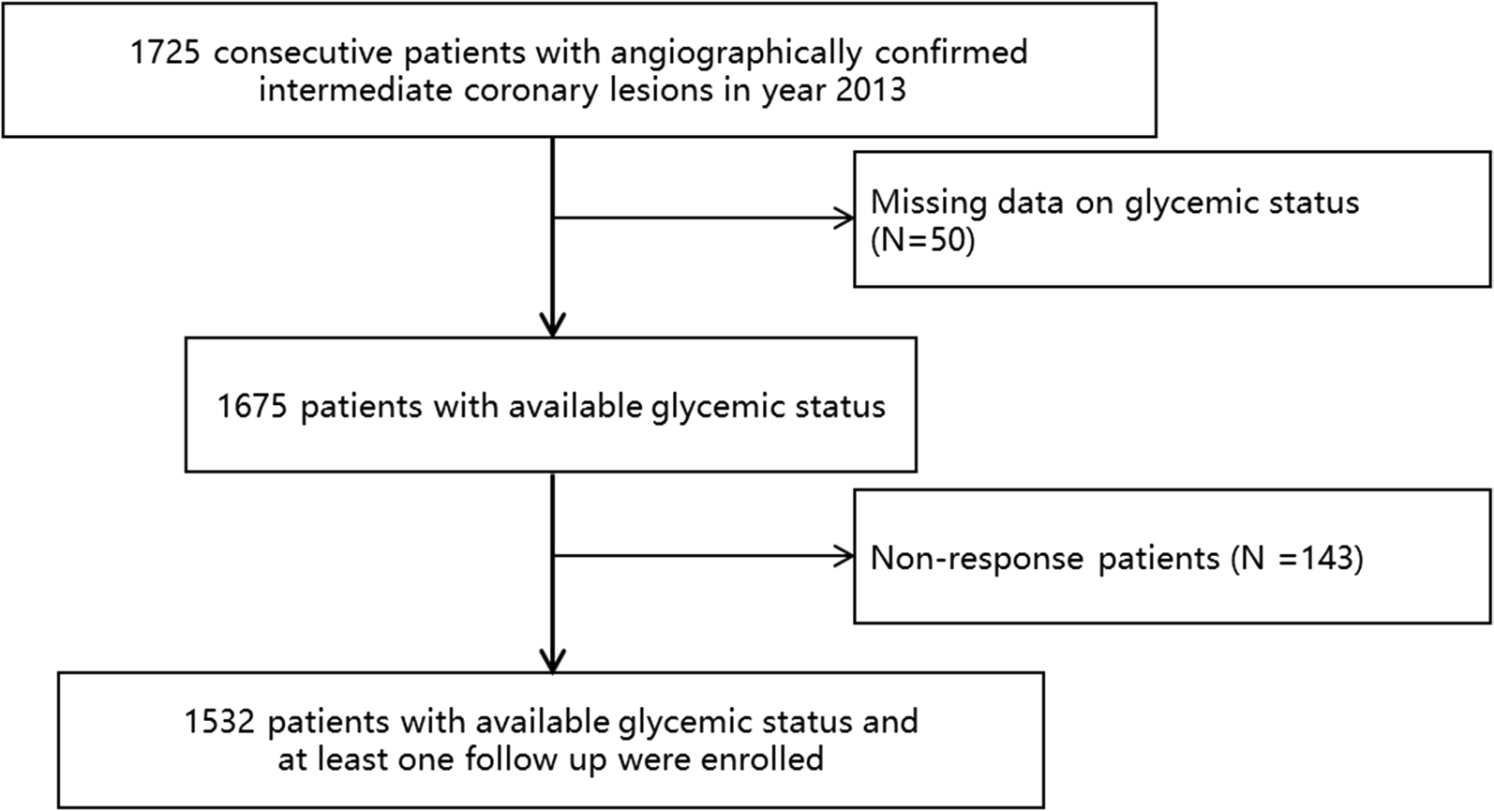
Study flow chart. A total of 1725 consecutive patients with angiographically confirmed intermediate coronary lesions were enrolled in year 2013. After excluding 50 patients with missing data on glycemic status and 143 patients who did not response to our follow-up invitation, the current study included a total of 1532 patients

From January 2013 to February 2013, a total of 1725 consecutive patients who.

had coronary angiographically confirmed intermediate lesions were admitted to Fuwai Hospital. We excluded a total of 50 patients with missing data on glycemic status and 143 patients who did not response to our follow-up invitation, and finally included a total of 1532 patients (Fig. 1).

**Fig. 2:**
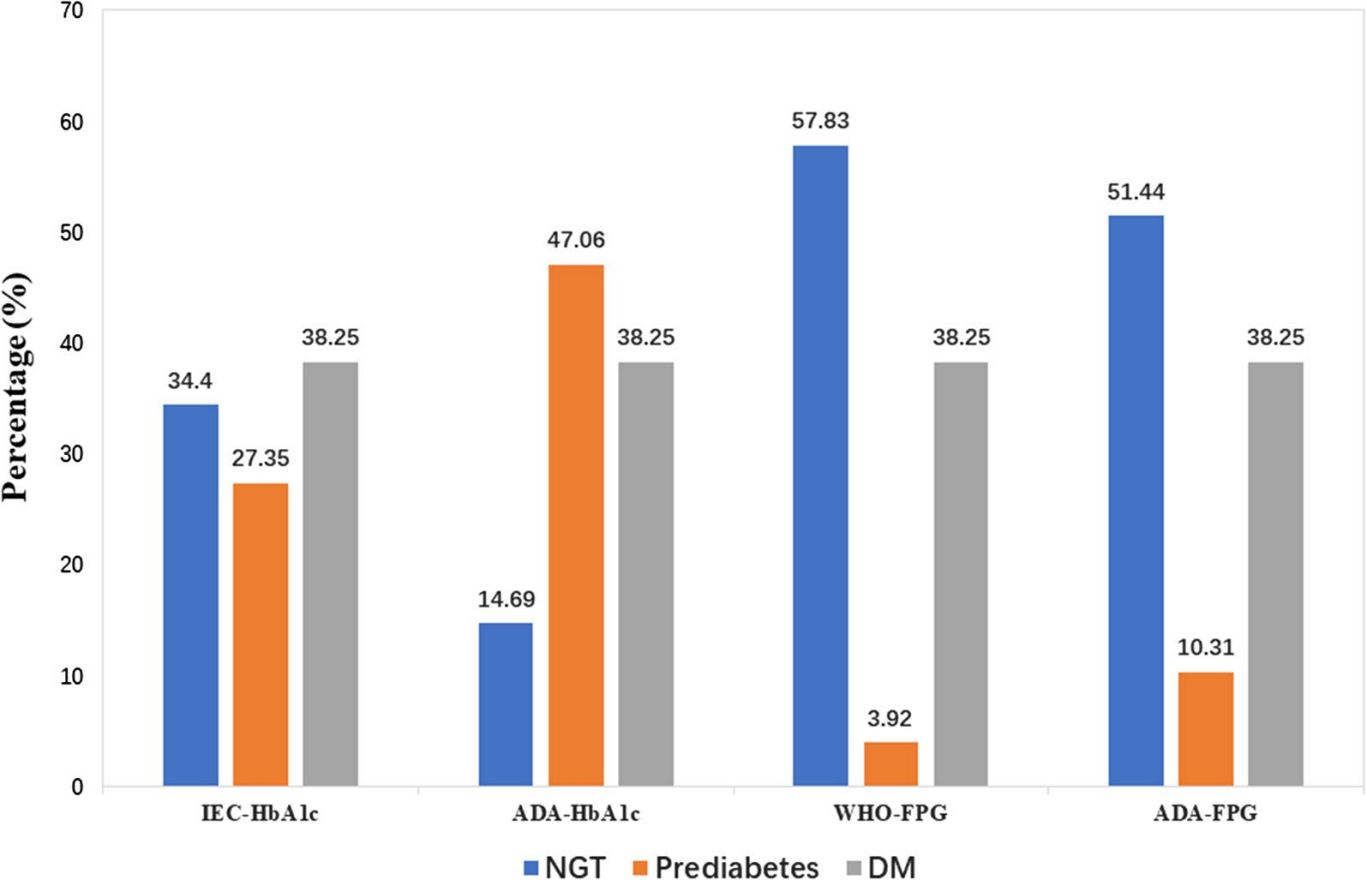
The percentage of patients according to categories of abnormal glucose metabolism by various definitions

The percentage of patients according to categories of abnormal glucose metabolism based on various definition are shown in Fig. 2. The number (proportion) of patients who NGT according to the IEC HbA1c-, ADA HbA1c -, ADA FPG- and WHO FPG- based definition were 527 (34.40%), 225 (14.69%), 788 (51.44%), and 886 (57.83%) respectively. The number (proportion) of patients who had prediabetes according to the IEC HbA1c-, ADA HbA1c—ADA FPG- and WHO FPG-based definition 419 (27.35%), 721 (47.06%), 158 (10.31%), 60 (3.92%) respectively.

### Baseline characteristics according to categories of abnormal glucose metabolism

Baseline characteristics according to the IEC HbA1c-based definition is shown in. Table 2. Compared with those with abnormal glucose metabolism, patients with normal glucose tolerance were younger, had lower BMI and heart rate. The proportion of patients with hypertension and hyperlipidemia were lower in the NGT group compared with abnormal glucose metabolism groups. Patients who had NGT had lower hsCRP, D-Dimer. No significant difference in smoking and alcohol status, as well as the proportion of triple vessel disease were found across groups. Baseline characteristics according to other abnormal glucose metabolism definitions are shown in Additional file 1: Tables S2, S3, S4.

**Table 2:** Baseline characteristics according to categories of abnormal glucose metabolism based on IEC HbA1c-based definition

Variables	NGT N = 527	Prediabetes N = 419	DM N = 586	P value
Age (years)	56.68 ± 9.93	60.63 ± 9.49	60.47 ± 9.14	< 0.0001
Female (%)	150/527 (28.46)	129/419 (30.79)	209/586 (35.67)	0.0312
Hypertension (%)	304/527 (57.69)	272/419 (64.92)	437/586 (74.57)	< 0.0001
Hyperlipidemia (%)	270/527 (51.23)	263/419 (62.77)	382/586 (65.19)	< 0.0001
Smoke (%)	233/527 (44.21)	170/419 (40.57)	265/586 (45.22)	0.3216
Alcohol (%)	254/527 (48.20)	190/419 (45.35)	252/586 (43.00)	0.2208
BMI (kg/m^2^)	25.18 ± 3.02	25.73 ± 3.07	26.22 ± 3.26	< 0.0001
HR (bpm)	68.66 ± 9.69	69.62 ± 10.38	71.30 ± 12.15	0.0002
SBP (mmHg)	127.09 ± 16.24	126.45 ± 16.40	129.03 ± 16.37	0.0624
LVEF (%)	64.77 ± 5.88	65.10 ± 6.68	64.45 ± 6.50	0.4097
NT-proBNP (pmol/L)	537.40 (427.40, 693.90)	550.95 (441.90, 703.20)	547.15 (432.75, 737.35)	0.4443
hsCRP (mg/L)	1.01 (0.54, 1.88)	1.36 (0.67, 2.64)	1.56 (0.80, 3.08)	< 0.0001
Cr (umol/L)	72.94 (63.17, 82.35)	73.25 (64.00, 80.95)	72.60 (62.62, 82.38)	0.8906
D_Dimer (ug/ml)	0.26 (0.18, 0.37)	0.28 (0.20, 0.39)	0.29 (0.20, 0.41)	0.0005
TC (mmol/L)	4.22 (3.56, 4.87)	4.14 (3.48, 4.86)	4.04 (3.38, 4.78)	0.1502
LDL (mmol/L)	2.46 (1.93, 3.10)	2.42 (1.80, 3.08)	2.32 (1.84, 3.00)	0.0953
HDL (mmol/L)	1.10 (0.90, 1.30)	1.08 (0.90, 1.28)	1.02 (0.86, 1.20)	< 0.0001
Lpa (mg/L)	162.36 (65.18, 320.46)	150.19 (64.42, 334.84)	145.11 (53.53, 358.73)	0.5735
Fasting glucose (mmol/L)	5.03 ± 0.55	5.26 ± 0.57	7.12 ± 2.60	< 0.0001
HbA1c (%)	5.64 ± 0.24	6.16 ± 0.13	7.26 ± 1.23	< 0.0001
Angiographic characteristics				
LM (%)	21/527 (3.98)	17/419 (4.06)	16/586 (2.73)	0.4138
RCA (%)	119/527 (22.58)	82/419 (19.57)	146/586 (24.91)	0.1364
LAD (%)	349/527 (66.22)	254/419 (60.62)	388/586 (66.21)	0.1240
LCX (%)	122/527 (23.15)	107/419 (25.54)	196/586 (33.45)	0.0003
Triple-vessel disease	23/527 (4.36)	9/419 (2.15)	30/586 (5.12)	0.0560

*NGT*  normal glucose tolerance, *DM*  diabetes mellitus, *BMI* body mass index, *HR* heart rate, *SBP* systolic blood pressure, *LVEF* left ventricular ejection fraction, *NT-proBNP* N-Terminal Pro–B-Type Natriuretic Peptide, *hsCRP* high-sensitivity C-reactive protein, *Cr* Creatinine, *TC* Total cholesterol, *LDL* low-density lipoprotein, *HDL* high-density lipoprotein, *Lpa*  Lipoprotein (a), *HbA1c* glycated hemoglobin, *LM * left main; *RCA*  right coronary artery, *LAD*  left anterior descending, *LCX*  left circumflex artery

### Association between abnormal glucose metabolism and long-term outcome

**Fig. 3:**
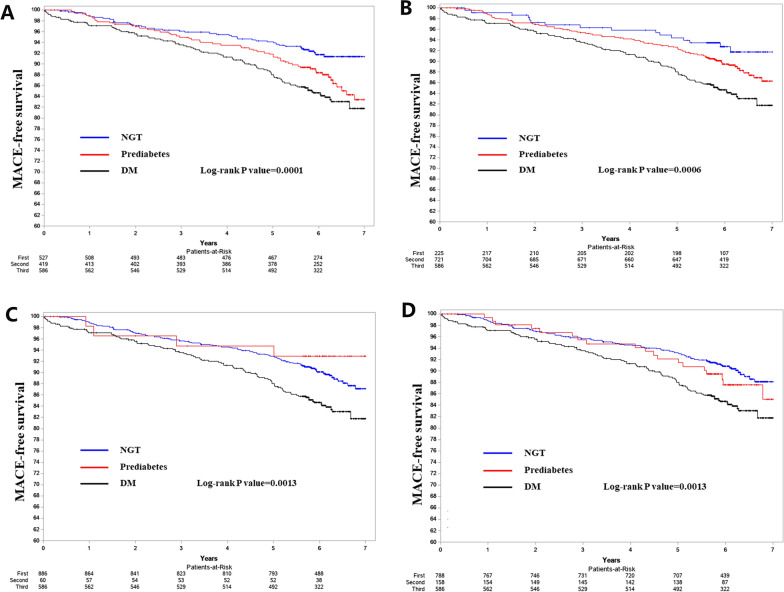
Kaplan–Meier curve showing survival free of MACE for different categories of abnormal glycemic metabolism according to IEC HbA1c-(**A**), ADA HbA1c- (**B**), ADA FPG- (**C**) and WHO FPG- (**D**) based definition

A total of 197 MACE occurred during a median follow-up time of 6.1 years, and. Included 62 deaths, 31 MI and 125 revascularization (a total of 21 patients suffered from both MI and revascularization). According to the IEC HbA1c-based definition, a total of 41 (7.78%) events occurred in the NGT group, 58 (13.84%) events occurred in the prediabetes group, 98 (16.72%) events occurred in the DM group (Table 3). Compared with normal glucose metabolism, each category of abnormal glucose metabolism was associated with higher risk of MACE. Compared with the NGT group, the HR (95% CI) of MACE was 1.705 (1.143, 2.543) for the prediabetes group, 2.173 (1.509, 3.129) for the DM group. The Kaplan–Meier curves showing the survival freedom from the MACE across groups are shown in Fig. 3A. The multivariate-adjusted HR (95% CI) for MACE was 1.513 (1.005, 2.277) for the prediabetes 1.870 (1.273, 2.745) for the DM group. The number and proportion of events according to categories of abnormal glucose metabolism based on other definitions are shown in Additional file 1: Tables S5, S6, S7. In general, prediabetes was not significantly associated with MACE risk, and DM was associated with increased MACE risk according to the ADA HbA1c—ADA FPG-, and WHO FPG-based definition. The Kaplan–Meier curves showing the survival freedom from the MACE across groups are shown in Fig. 3B, D.

**Table 3:** Adjusted HR for MACE during 6-year follow-up according to baseline categories of abnormal glucose metabolism by IEC HbA1c-based definition

	Event/Total (%)	HR (95% CI)	P value
Model 1			
NGT	41/527 (7.78)	1 (reference)	1 (reference)
Prediabetes	58/419 (13.84)	1.705 (1.143, 2.543)	0.0090
DM	98/586 (16.72)	2.173 (1.509, 3.129)	< .0001
Model 2			
NGT	41/527 (7.78)	1 (reference)	1 (reference)
Prediabetes	58/419 (13.84)	1.514 (1.011, 2.267)	0.0440
DM	98/586 (16.72)	2.027 (1.403, 2.927)	0.0002
Model 3			
NGT	41/527 (7.78)	1 (reference)	1 (reference)
Prediabetes	58/419 (13.84)	1.513 (1.005, 2.277)	0.0471
DM	98/586 (16.72)	1.870 (1.273, 2.745)	0.0014

Model 1 is univariate analysis

Model 2 adjusted for age and sex

Model 3 adjusted for model 2 plus medical history of hypertension, hyperlipidemia, smoking status, alcoholic consumption, body mass index, heart rate, total cholesterol, LDL, HDL, hsCRP, D-Dimer and triple vessel disease;

*MACE* major adverse cardiovascular events, *IEC*  international expert committee, *HbA1C* glycated haemoglobin, *NGT* normal glucose tolerance, *DM* diabetes mellitus, *HR* hazard ratio, *CI* confidence interva

### The association between glycemic parameters and MACE

We next investigated the relationship between glycemic parameters (HbA1c and admission fasting glucose) as a continuous variable and outcome. Restricted cubic spline showed that HbA1c presented a linear relationship with the risk of MACE (p for non-linearity = 0.2119), and the risk of MACE increased along with HbA1c levels (Additional file 1: Fig. S1A). Admission fasting glucose also presented a linear relationship with the risk of MACE (p for non-linearity = 0.4014), but a significant increased risk along with fasting glucose level was not observed (Additional file 1: Fig. S1B). When glycemic parameters were modelled as a continuous variable, the HR (95% CI) for MACE was 2.150 (1.124, 4.115) per doubling increase in HbA1c (Additional file 1: Table S8), and 1.021 (0.952, 1.094) per unit increase in admission fasting glucose in the fully adjusted model (Additional file 1: Table S9). The best cut-off point of HbA1c based on Youden’s index was 6.0% in predicting MACE in patients without known diabetes, with sensitivity of 0.667 and specificity of 0.493 (Additional file 1: Fig. S2).

The correlation between HbA1c and hsCRP, N-Terminal Pro–B-Type Natriuretic Peptide (NT-proBNP), LDL, triglyceride, total cholesterol, HDL and fasting plasma glucose are shown in Additional file 1: Fig. S3. HbA1c level was positively associated with hsCRP (R^2^ = 0.1917, p < 0.0001), triglycerides (R^2^ = 0.0927, p = 0.0003), FPG (R^2^ = 0.5434, p < 0.0001) and negatively associated with HDL (R^2^ = − 0.0862, P = 0.0009). No significant association between HbA1c and NT-proBNP, LDL, and total cholesterol was observed. Of note, the correlation coefficient was weak despite statistically significant, and may not be able to provide sufficient clinical significance.

### Subgroup analysis of the association between abnormal glucose metabolism and long-term outcome

Subgroup analysis of the association between abnormal glucose metabolism based on IEC HbA1c-based definition with MACE according to age, sex, smoking status and medical history of hypertension are shown in Additional file 1: Table S10. P value for interaction was greater than 0.05 across all subgroup analyses, indicating that the effect of categories of abnormal remains consistent patients according to age (age ≥ 65 years or age < 65 years), sex (female or male subgroup), smoking status (current smokers or nonsmokers) and medical history of hypertension (with or without medical history of hypertension).

## Discussion

### Major findings

By investigating the association between categories of abnormal glucose.

metabolism based on various definition and MACE in patients with intermediate lesions, the current study found that prediabetes based on IEC HbA1c-based definition (6.0 ≤ HbA1c < 6.5%) was associated with significant increased MACE risk compared with NGT, which was consistent with the best cut-off point identified based on the Youden’s index. Newly diagnosed diabetes was associated with increased MACE risk compared with normal glycemia based on all the currently widely used definitions. Globally, FPG or FPG-based definition of prediabetes was not associated with patients’ outcome. The current study supported the use of IEC HbA1c-based definition to identify high-risk patients of MACE, who may benefit from early lifestyle interventions.

### Reasons for selecting patients with intermediate lesions

The current study enrolled patients with angiographically confirmed coronary.

intermediate lesions to represent patients with stable coronary heart disease for the following two reasons: On the one hand, patients with coronary intermediate lesions had similar degree of coronary stenoses (DS% of 50–70%), and thus the effect of lesion stenosis severity on prognosis may be reduced. On the other hand, patients with coronary intermediate lesions various significantly in short-term prognosis. In patients without functional significant lesions and deferred from revascularization therapy, MACE occurred in approximately 4% of the population in one-year follow-up [10], suggesting that further investigation of prognostic factors will assist in risk stratification and outcome improvement.

### Explanations for the superiority of HbA1c over FPG

Our findings showed that prediabetes defined based on HbA1c, but not fasting plasma glucose, identified a group of patients at high-risk of MACE. Explanations for the superiority of HbA1c over fasting glucose to identify patients at risk for MACE are proposed as follows: Glycated hemoglobin values reflect the three-month average endogenous exposure to glucose, including postprandial spikes, and show low intraindividual variability, particularly in people without diabetes [11]. In addition, HbA1c is a useful marker for other glycated molecules, such as advanced glycation end-products, which are likely drivers of vascular inflammation and subsequent plaque progression and rupture, leading to major adverse events in patients [12]. These features support the role of HbA1c as a novel biomarker in risk stratification.

### Comparison with previous studies

Growing number of studies explored the association between prediabetes defined based on HbA1c value and MACE in the setting of CAD [1]. However, most studies enrolled patients with acute coronary syndrome [13, 14] or those who received revascularization [14–16], and the current study add new data in this field by examining this association in patients with stable CAD and not undergoing revascularization. Our study found that prediabetes defined according to IEC HbA1c-based definition was associated with increased risk of MACE. Our findings are in consistent with previous studies showing that prediabetes was associated with poorer prognosis in patients who underwent PCI and treated with contemporary drug eluting stents (DES) [16, 17]. In contrast, some previous studies reported no significant association between HbA1c-defined prediabetes and prognosis [15, 18]. The contradictory results may be explained by the difference in prediabetes definition and endpoint. In the above two studies, prediabetes was defined by HbA1c-ADA definition, which is HbA1c of 5.7–6.4%. Similarly, when prediabetes was defined based on HbA1c-ADA definition in our study, approximately half of the study population were classified as prediabetes, and only 15% of the study population were classified as normal glucose metabolism. This may explain the non-significant association between prediabetes and outcome.

### Possible underlying mechanisms

Several plausible biological mechanisms have been proposed to explain a possible direct relationship between chronically elevated blood glucose levels and coronary heart disease (CHD) [24]. Glucose can react with many different proteins, creating advanced glycation end products (AGE), which contribute to long-term complications in diabetes as well as to endothelial dysfunction, plaque formation and progression.

[19] reported that circulating AGEs and soluble receptor for AGE (RAGE) isoforms in patients with type 2 diabetes as predictors of MACE and all-cause mortality [19]. In addition, AGEs can be estimated by the non-invasive skin autofluorescence, and provided additional prognostic information in patients with both type 1 [20] and type 2 diabetes [21]. In addition to the direct effect of elevated glucose on atherosclerosis, chronically elevated blood glucose levels, as reflected by greater HbA1c level, is also related to increased risk of other CHD risk factors including diabetic dyslipidemia [22], hypertension [23], which together accelerate vascular injury and cardiovascular disease risk [24]. Diabetes has been proposed to accelerate atherosclerosis via oxidative stress, and increased inflammation [25].

### Clinical significance

Our study suggested that prediabetes based on the IEC HbA1c-based definition predicts MACE risk in patients with stable CAD. As discussed above, HbA1c reflects the average endogenous exposure to glucose and have low variability compared with fasting glucose. In addition, it less time-consuming compared with OGTT. These characteristics may contribute to the superiority of glycated hemoglobin over other diagnostic methods for long-term risk stratification. Of note, newly diagnosed diabetes across all the four definitions was associated with a significant increased MACE risk compared with normal glycemic metabolism. Since patients with prediabetes have a significant higher risk of progression to diabetes, efforts including dietary and exercise intervention should be made in all patients with CAD and abnormal glucose metabolism [26]. Regarding pharmacologic interventions, no pharmacologic agent has been approved currently by the U.S. Food and Drug administration specifically for diabetes prevention or the treatment for prediabetes [27]. However, recent cardiovascular outcomes trials indicated cardiovascular benefits of novel glucose-lowering drugs, which included sodium-glucose cotransporter-2 inhibitors and glucagon-like peptide-1 receptor agonists. Therefore, these drugs may be recommended in patients with prediabetes to prevent or delay the onset of diabetes, which requires validation in future studies [28].

### Limitations

Our study has several limitations: Firstly, OGTT tests were not performed in the majority of patients, and the relationship between post-load glucose value, and impaired glucose tolerance, another form of prediabetes defined by the 2 h-postload glucose level was not evaluated. Secondly, only baseline HbA1c was collected, while the association between variations in HbA1c during follow-up was not assessed. Finally, the current study was a single center study with moderate size, and unmeasured confounders could not be excluded. Our findings need further validation in large-scale prospective cohort in future studies.

## Conclusions

The prevalence of prediabetes varies significantly according to different definitions, and a high proportion of patients with coronary intermediate lesions without previously known history of diabetes have abnormal glycemic metabolism, suggesting the importance of screening for diabetes in this population. In our study cohort, prediabetes according to IEC HbA1c-based definition was associated with significant increased MACE risk compared with NGT, and newly diagnosed diabetes was associated with increased MACE risk based on all the currently widely used definitions. The current study supported the use of IEC HbA1c-based definition to identify high-risk patients of MACE, who may benefit from early lifestyle interventions, and these findings require further validation in future studies.

## Supplementary Information

**Additional file 1: Table S1.** MACE risk according to baseline variables. **Table S2.** Baseline characteristics according to categories of abnormal glucose metabolism based on ADA HbA1c-based definition. **Table S3**. Baseline characteristics according to categories of abnormal glucose metabolism based on ADA FPG-based definition. **Table S4.** Baseline characteristics according to categories of abnormal glucose metabolism based on WHO FPG-based definition. **Table S5.** Adjusted HR for MACE during 6-year follow-up according to baseline categories of abnormal glucose metabolism by ADA HbA1c-based definition. **Table S6.** Adjusted HR for MACE during 6 year follow-up according to baseline categories of abnormal glucose metabolism by WHO FPG-based definition. **Table S7.** Adjusted HR for MACE during 6 year follow-up according to baseline categories of abnormal glucose metabolism by ADA FPG-based definition. **Table S8.** Adjusted HR for MACE during 6 year follow-up according to baseline HbA1c level as a continuous variable (log2 transformed). **Table S9.** Adjusted HR for MACE during 6 year follow-up according to baseline glucose level as a continuous variabl. **Table S10.** Subgroup analysis of the association between categories of abnormal glucose metabolism and MACE. **Figure S1.** Restricted cubic spline analysis of the association between baseline HbA1c level (**A**) and admission fasting glucose (**B**) and major cardiovascular event (MACE) risk. Baseline HbA1c and admission fasting glucose level presented a linear relationship with the risk of MACE (p for non-linearity 0.2119 and 0.4014 respectively). The curves are presented with 95% confidence interval. **Figure S2.** ROC Curve of HbA1c in Predicting MACE. The c-index on the basis of the AUC for HbA1c in predicting ischemic stroke was 0.5927. The best cutoff value of HbA1c based on the highest Youden’s index was 6% with sensitivity of 0.667 and specificity of 0.493. **Figure S3.** Correlation analysis of the relationship between HbA1c and hsCRP (**A**), NT-proBNP (**B**), LDL (**C**), triglyceride (**D**), total cholesterol (**E**), HDL (**F**) and fasting glucose (**G**).

## Data Availability

Data is available upon reasonable request to the corresponding author.
