# Supplementary material for: HbA1c-based rather than fasting plasma glucose-based definitions of prediabetes identifies high-risk patients with angiographic coronary intermediate lesions: a prospective cohort study
Source: Cardiovasc Diabetol. 2023 Mar 25;22:68. doi: 10.1186/s12933-023-01750-6 (PMC10040102; doi:10.1186/s12933-023-01750-6)
Supplement: Supplementary file 1 — Additional file 1: Table S1. MACE risk according to baseline variables. Table S2. Baseline characteristics according to categories of abnormal glucose metabolism based on ADA HbA1c-based definition. Table S3. Baseline characteristics according to categories of abnormal glucose metabolism based on ADA FPG-based definition. Table S4. Baseline characteristics according to categories of abnormal glucose metabolism based on WHO FPG-based definition. Table S5. Adjusted HR for MACE during 6-year follow-up according to baseline categories of abnormal glucose metabolism by ADA HbA1c-based definition. Table S6. Adjusted HR for MACE during 6 year follow-up according to baseline categories of abnormal glucose metabolism by WHO FPG-based definition. Table S7. Adjusted HR for MACE during 6 year follow-up according to baseline categories of abnormal glucose metabolism by ADA FPG-based definition. Table S8. Adjusted HR for MACE during 6 year follow-up according to baseline HbA1c level as a continuous variable (log2 transformed). Table S9. Adjusted HR for MACE during 6 year follow-up according to baseline glucose level as a continuous variabl. Table S10. Subgroup analysis of the association between categories of abnormal glucose metabolism and MACE. Figure S1. Restricted cubic spline analysis of the association between baseline HbA1c level (A) and admission fasting glucose (B) and major cardiovascular event (MACE) risk. Baseline HbA1c and admission fasting glucose level presented a linear relationship with the risk of MACE (p for non-linearity 0.2119 and 0.4014 respectively). The curves are presented with 95% confidence interval. Figure S2. ROC Curve of HbA1c in Predicting MACE. The c-index on the basis of the AUC for HbA1c in predicting ischemic stroke was 0.5927. The best cutoff value of HbA1c based on the highest Youden’s index was 6% with sensitivity of 0.667 and specificity of 0.493. Figure S3. Correlation analysis of the relationship between HbA1c and hsCRP (A), NT-proBNP (B), [file 12933_2023_1750_MOESM1_ESM.docx]

**Additional file 1: TableS1. MACE risk according to baseline variables**

|  | HR (95%CI) | P |
| --- | --- | --- |
| Age (per one year increase) | 1.031 (1.017, 1.046) | <.0001 |
| Sex (female as reference) | 1.547 (1.115, 2.147) | 0.0091 |
| BMI (per 1 kg/m^2^ increase) | 1.039 (0.994, 1.086) | 0.0922 |
| Heart rate (per 1 bpm) | 0.994 (0.981, 1.007) | 0.3793 |
| SBP (per 1 mmHg increase) | 1.003 (0.995, 1.012) | 0.4674 |
| LVEF (per one percentage increase) | 0.983 (0.959, 1.008) | 0.1750 |
| BNP (per doubling increase) | 1.170 (0.939, 1.456) | 0.1611 |
| HsCRP (per 1 mg/L increase) | 1.030 (0.991, 1.070) | 0.1328 |
| Cr (per 1 umol/L increase) | 1.000 (0.998, 1.001) | 0.7090 |
| D-Dimer (per 1 ug/ml increase) | 1.058 (0.927, 1.208) | 0.4018 |
| HbA1C (per 1% increase) | 1.159 (1.041, 1.291) | 0.0069 |
| Fasting glucose (per 1 mg/L increase) | 1.031 (0.968, 1.099) | 0.3398 |
| TC (per 1 mmol/L increase) | 0.918 (0.803, 1.049) | 0.2106 |
| LDL (per 1 mmol/L increase) | 0.933 (0.794, 1.096) | 0.3955 |
| HDL (per 1 mmol/L increase) | 0.553 (0.338, 0.904) | 0.0182 |
| Lpa (per 1 mg/L increase) | 1.000 (0.999, 1.001) | 0.9097 |
| Diabetes (no as reference) | 1.306 (0.955, 1.786) | 0.0948 |
| Hypertension (no as reference) | 1.276 (0.938, 1.737) | 0.1207 |
| Hyperlipidemia (no as reference) | 1.133 (0.849, 1.512) | 0.3967 |
| Smoke (no as reference) | 1.545 (1.168, 2.045) | 0.0023 |
| Drink (no as reference) | 1.423 (1.075, 1.883) | 0.0136 |
| LM (no as reference) | 1.194 (0.588, 2.424) | 0.6235 |
| RCA (no as reference) | 1.535 (1.130, 2.086) | 0.0061 |
| LAD (no as reference) | 0.956 (0.715, 1.277) | 0.7602 |
| LCX (no as reference) | 1.759 (1.320, 2.343) | 0.0001 |
| Triple-vessel disease | 2.188 (1.292, 3.705) | 0.0036 |

Abbreviations are the same as table 1.

**Additional file 1: TableS2. Baseline characteristics according to categories of abnormal glucose metabolism based on ADA HbA1c-based definition**

| Variables | NGT  N=225 | Pre-diabetes  N=721 | DM  N=586 | P value |
| --- | --- | --- | --- | --- |
| Age (years) | 56.02±10.21 | 59.50±9.60 | 60.47±9.14 | <.0001 |
| Female (%) | 55/225(24.44) | 224/721 (31.07) | 209/586 (35.67) | 0.0074 |
| Hypertension (%) | 137/225 (60.89) | 439/721 (60.89) | 437/586 (74.57) | <.0001 |
| Hyperlipidemia (%) | 116/225 (51.56) | 417/721 (57.84) | 382/586 (65.19) | 0.0007 |
| Smoke (%) | 98/225 (43.56) | 305/721 (42.30) | 265/586 (45.22) | 0.5710 |
| Alcohol (%) | 113/225 (50.22) | 331/721 (45.91) | 252/586 (43.00) | 0.1701 |
| BMI (kg/m^2^) | 25.48±2.85 | 25.41±3.12 | 26.22±3.26 | <.0001 |
| HR (bpm) | 69.38±9.58 | 68.99±10.14 | 71.30±12.15 | 0.0005 |
| SBP (mmHg) | 126.31±15.48 | 126.96±16.56 | 129.03±16.37 | 0.0317 |
| LVEF (%) | 64.44±5.35 | 65.06±6.50 | 64.45±6.50 | 0.3149 |
| NT-proBNP (pmol/L) | 537.40  (425.20, 672.80) | 544.40  (431.50, 714.00) | 547.15  (432.75, 737.35) | 0.4503 |
| hsCRP (mg/L) | 0.93 (0.53, 1.80) | 1.19 (0.63, 2.32) | 1.56 (0.80, 3.08) | <.0001 |
| Cr (umol/L) | 72.04  (61.38, 80.62) | 73.55  (64.28, 81.86) | 72.60  (62.62, 82.38) | 0.4844 |
| D_Dimer (ug/ml) | 0.24 (0.18, 0.35) | 0.27 (0.19, 0.38) | 0.29 (0.20, 0.41) | 0.0039 |
| TC (mmol/L) | 4.19 (3.57, 4.79) | 4.19 (3.50, 4.89) | 4.04 (3.38, 4.78) | 0.1972 |
| LDL (mmol/L) | 2.49 (1.88, 3.09) | 2.43 (1.83, 3.10) | 2.32 (1.84, 3.00) | 0.1777 |
| HDL (mmol/L) | 1.11 (0.94, 1.31) | 1.08 (0.89, 1.29) | 1.02 (0.86, 1.20) | <.0001 |
| Lpa (mg/L) | 161.13  (64.15, 356.19) | 155.38  (64.91, 319.91) | 145.11  (53.53, 358.73) | 0.4510 |
| Fasting glucose (mg/L) | 4.96±0.53 | 5.18±0.58 | 7.12±2.60 | <.0001 |
| HbA1c (%) | 5.41±0.20 | 6.01±0.21 | 7.26±1.23 | <.0001 |
| Angiographic characteristics |  |  |  |  |
| LM (%) | 9/225 (4.00) | 29/721 (4.02) | 15/586 (2.73) | 0.4145 |
| RCA (%) | 48/225 (21.33) | 153/721 (21.22) | 146/586 (24.91) | 0.2492 |
| LAD (%) | 162/225 (72.00) | 441/721 (61.17) | 388/586 (66.21) | 0.0075 |
| LCX (%) | 45/225(20.00) | 184/721 (25.52) | 196/586 (33.45) | 0.0001 |
| Triple-vessel disease | 11/225 (4.89) | 21/721 (2.91) | 30/586 (5.12) | 0.1035 |

Abbreviations are the same as table 1.

**Additional file 1: TableS3. Baseline characteristics according to categories of abnormal glucose metabolism based on ADA FPG-based definition**

| Variables | NGT  N=788 | Pre-diabetes  N=158 | DM  N=586 | P value |
| --- | --- | --- | --- | --- |
| Age (years) | 58.05±10.02 | 60.33±9.26 | 60.47±9.14 | <.0001 |
| Female (%) | 237/788 (30.08) | 42/158 (26.58) | 209/586 (35.67) | 0.0288 |
| Hypertension (%) | 459/788 (58.25) | 117/158 (74.05) | 437/586 (74.57) | <.0001 |
| Hyperlipidemia (%) | 427/788 (54.19) | 106/158 (67.09) | 382/586 (65.19) | <.0001 |
| Smoke (%) | 331/788 (42.01) | 72/158 (45.57) | 265/586 (45.22) | 0.4293 |
| Alcohol (%) | 371/788 (47.08) | 73/158 (46.20) | 252/586 (43.00) | 0.3172 |
| BMI (kg/m^2^) | 25.39±3.07 | 25.58±3.02 | 26.22±3.26 | <.0001 |
| HR (bpm) | 6855±9.72 | 71.73±11.00 | 71.30±12.15 | <.0001 |
| SBP (mmHg) | 125.97±15.88 | 131.01±17.74 | 129.03±16.37 | <.0001 |
| LVEF (%) | 64.94±6.21 | 64.87±6.66 | 64.45±6.50 | 0.5020 |
| NT-proBNP (pmol/L) | 539.75  (428.40, 703.10) | 569.50  (461.80, 683.10) | 547.15  (432.75, 737.35) | 0.3770 |
| hsCRP (mg/L) | 1.09 (0.59, 2.17) | 1.38 (0.70, 3.04) | 1.56 (0.80, 3.08) | <.0001 |
| Cr (umol/L) | 73.06  (63.08, 81.39) | 72.67  (64.90, 81.79) | 72.60  (62.62, 82.38) | 0.9430 |
| D_Dimer (ug/ml) | 0.26 (0.19, 0.37) | 0.29 (0.19, 0.40) | 0.29 (0.20, 0.41) | 0.0136 |
| TC (mmol/L) | 4.15 (3.47, 4.83) | 4.37 (3.74, 5.11) | 4.04 (3.38, 4.78) | 0.0117 |
| LDL (mmol/L) | 2.43 (1.83, 3.06) | 2.54 (1.92, 3.26) | 2.32 (1.84, 3.00) | 0.0601 |
| HDL (mmol/L) | 1.08 (0.89, 1.29) | 1.11 (0.95, 1.32) | 1.02 (0.86, 1.20) | <.0001 |
| Lpa (mg/L) | 160.84  (72.11, 331.93) | 140.08  (48.76, 303.56) | 145.11  (53.53, 358.73) | 0.1232 |
| Fasting glucose (mg/L) | 4.95±0.42 | 6.04±0.28 | 7.12±2.60 | <.0001 |
| HbA1c (%) | 5.84±0.32 | 6.00±0.31 | 7.26±1.23 | <.0001 |
| Angiographic characteristics |  |  |  |  |
| LM (%) | 33/788 (4.19) | 5/158 (3.16) | 15/586 (2.73) | 0.3385 |
| RCA (%) | 162/788 (20.56) | 39/158 (24.68) | 146/586 (24.91) | 0.1316 |
| LAD (%) | 503/788 (63.83) | 100/158 (63.29) | 388/586 (66.21) | 0.6117 |
| LCX (%) | 191/788 (24.24) | 38/158 (24.05) | 196/586 (33.45) | 0.0004 |
| Triple-vessel disease | 27/788 (3.43) | 5/158 (3.16) | 30/586 (5.12) | 0.2424 |

Abbreviations are the same as table 1.

**Additional file 1: TableS4. Baseline characteristics according to categories of abnormal glucose metabolism based on WHO FPG-based definition**

| Variables | NGT  N=886 | Pre-diabetes  N=60 | DM  N=586 | P value |
| --- | --- | --- | --- | --- |
| Age (years) | 58.29±9.99 | 60.54±8.85 | 60.47±9.14 | <.0001 |
| Female (%) | 266/886 (30.02) | 13/60 (21.67) | 209/586 (35.67) | 0.0169 |
| Hypertension (%) | 529/886 (59.71) | 47/60 (78.33) | 437/586 (74.57) | <.0001 |
| Hyperlipidemia (%) | 496/886 (55.98) | 37/60 (61.67) | 382/586 (65.19) | 0.0019 |
| Smoke (%) | 372/886 (41.99) | 31/60 (51.67) | 265/586 (45.22) | 0.2067 |
| Alcohol (%) | 415/886 (46.84) | 29/60 (48.33) | 252/586 (43.00) | 0.3157 |
| BMI (kg/m^2^) | 25.18±3.04 | 25.92±3.32 | 26.22±3.26 | <.0001 |
| HR (bpm) | 68.75±9.83 | 73.97±11.29 | 71.30±12.15 | <.0001 |
| SBP (mmHg) | 126.56±16.29 | 130.48±16.09 | 129.03±16.37 | 0.0072 |
| LVEF (%) | 64.95±6.27 | 64.56±6.54 | 64.45±6.50 | 0.4714 |
| NT-proBNP (pmol/L) | 542.60 (431.50, 702.90) | 500.20 (411.80, 684.50) | 547.15  (432.75, 737.35) | 0.5886 |
| hsCRP (mg/L) | 1.11 (0.60, 2.20) | 1.56 (0.74, 4.55) | 1.56 (0.80, 3.08) | <.0001 |
| Cr (umol/L) | 74.43  (66.45, 84.55) | 73.66  (64.37, 82.78) | 72.60  (62.62, 82.38) | 0.3967 |
| D_Dimer (ug/ml) | 0.32 (0.24, 0.45) | 0.30 (0.20, 0.45) | 0.29 (0.20, 0.41) | 0.0016 |
| TC (mmol/L) | 4.17 (3.50, 4.85) | 4.39 (3.88, 5.24) | 4.04 (3.38, 4.78) | 0.0249 |
| LDL (mmol/L) | 2.43 (1.83, 3.09) | 2.62 (2.08, 3.21) | 2.32 (1.84, 3.00) | 0.0578 |
| HDL (mmol/L) | 1.09 (0.90, 1.29) | 1.14 (0.96, 1.36) | 1.02 (0.86, 1.20) | <.0001 |
| Lpa (mg/L) | 159.18  (67.36, 330.82) | 136.32  (48.00, 292.38) | 145.11  (53.53, 358.73) | 0.4024 |
| Fasting glucose (mg/L) | 5.05±0.49 | 6.35±0.20 | 7.12±2.60 | <.0001 |
| HbA1c (%) | 5.86±0.33 | 6.16±0.29 | 7.26±1.23 | <.0001 |
| Angiographic characteristics |  |  |  |  |
| LM (%) | 36/886 (4.06) | 2/60 (3.33) | 15/586 (2.56) | 0.3967 |
| RCA (%) | 186/886 (20.99) | 15/60 (25.00) | 146/586 (24.91) | 0.1928 |
| LAD (%) | 570/886 (64.33) | 33/60 (55.00) | 388/586 (66.21) | 0.2113 |
| LCX (%) | 211/886 (23.81) | 18/60 (30.00) | 196/586 (33.45) | 0.0003 |
| Triple-vessel disease | 30/886 (3.39) | 2/60 (3.33) | 30/586 (5.12) | 0.2452 |

Abbreviations are the same as table 1.

**Additional file 1: TableS5. Adjusted HR for MACE during 6-year follow-up according to baseline categories of abnormal glucose metabolism by ADA HbA1c-based definition**

|  | Event/Total (%) | HR (95% CI) | P value |
| --- | --- | --- | --- |
| Model 1 |  |  |  |
| NGT | 16/225 (7.11) | 1 (reference) | 1 (reference) |
| Prediabetes | 83/721 (11.51) | 1.564 (0.916, 2.670) | 0.1017 |
| DM | 98/586 (16.72) | 2.360 (1.391, 4.004) | 0.0015 |
| Model 2 |  |  |  |
| NGT | 16/225 (7.11) | 1 (reference) | 1 (reference) |
| Prediabetes | 83/721 (11.51) | 1.402 (0.818, 2.402) | 0.2189 |
| DM | 98/586 (16.72) | 2.135 (1.253, 3.639) | 0.0053 |
| Model 3 |  |  |  |
| NGT | 16/225 (7.11) | 1 (reference) | 1 (reference) |
| Prediabetes | 83/721 (11.51) | 1.404 (0.816, 2.415) | 0.2201 |
| DM | 98/586 (16.72) | 1.959 (1.135, 3.380) | 0.0157 |

Model 1 is univariate analysis;

Model 2 adjusted for age and sex;

Model 3 adjusted for model 2 plus medical history of hypertension, hyperlipidemia, smoking status, alcoholic consumption, body mass index, heart rate, total cholesterol, LDL, HDL, hsCRP, D-Dimer and triple vessel disease;

MACE = major adverse cardiovascular events; ADA =American Diabetes Association; HR = hazard ratio; CI =confidence interval; HbA1C = glycated haemoglobin; NGT=normal glucose tolerance; DM=diabetes mellitus

**Additional file 1: TableS6. Adjusted HR for MACE during 6-year follow-up according to baseline categories of abnormal glucose metabolism by WHO FPG-based definition**

|  | Event/Total (%) | HR (95% CI) | P value |
| --- | --- | --- | --- |
| Model 1 |  |  |  |
| NGT | 95/886 (10.72) | 1 (reference) | 1 (reference) |
| Prediabetes | 4/60 (6.67) | 0.619 (0.228, 1.685) | 0.3479 |
| DM | 98/586 (16.72) | 1.607 (1.212, 2.131) | 0.0010 |
| Model 2 |  |  |  |
| NGT | 95/886 (10.72) | 1 (reference) | 1 (reference) |
| Prediabetes | 4/60 (6.67) | 0.552 (0.203, 1.504) | 0.2453 |
| DM | 98/586 (16.72) | 1.569 (1.181, 2.082) | 0.0018 |
| Model 3 |  |  |  |
| NGT | 95/886 (10.72) | 1 (reference) | 1 (reference) |
| Prediabetes | 4/60 (6.67) | 0.583 (0.213, 1.594) | 0.2930 |
| DM | 98/586 (16.72) | 1.434 (1.065, 1.931) | 0.0174 |

Model 1 is univariate analysis;

Model 2 adjusted for age and sex;

Model 3 adjusted for model 2 plus medical history of hypertension, hyperlipidemia, smoking status, alcoholic consumption, body mass index, heart rate, total cholesterol, LDL, HDL, hsCRP, D-Dimer and triple vessel disease;

MACE = major adverse cardiovascular events; WHO = World Health Organization; FPG =fasting plasma glucose; HR = hazard ratio; CI =confidence interval; HbA1C = glycated haemoglobin; NGT=normal glucose tolerance; DM=diabetes mellitus

**Additional file 1: TableS7. Adjusted HR for MACE during 6-year follow-up according to baseline categories of abnormal glucose metabolism by ADA FPG-based definition**

|  | Event/Total (%) | HR (95% CI) | P value |
| --- | --- | --- | --- |
| Model 1 |  |  |  |
| NGT | 79/788 (10.03) | 1 (reference) | 1 (reference) |
| Prediabetes | 20/158 (12.66) | 1.274 (0.780, 2.080) | 0.3339 |
| DM | 98/586 (16.72) | 1.721 (1.280, 2.315) | 0.0003 |
| Model 2 |  |  |  |
| NGT | 79/788 (10.03) | 1 (reference) | 1 (reference) |
| Prediabetes | 20/158 (12.66) | 1.148 (0.702, 1.878) | 0.5818 |
| DM | 98/586 (16.72) | 1.663 (1.235, 2.240) | 0.0008 |
| Model 3 |  |  |  |
| NGT | 79/788 (10.03) | 1 (reference) | 1 (reference) |
| Prediabetes | 20/158 (12.66) | 1.200 (0.729, 1.975) | 0.4730 |
| DM | 98/586 (16.72) | 1.536 (1.124, 2.099) | 0.0071 |

Model 1 is univariate analysis;

Model 2 adjusted for age and sex;

Model 3 adjusted for model 2 plus medical history of hypertension, hyperlipidemia, smoking status, alcoholic consumption, body mass index, heart rate, total cholesterol, LDL, HDL, hsCRP, D-Dimer and triple vessel disease;

MACE = major adverse cardiovascular events; ADA =American Diabetes Association; HR = hazard ratio; CI =confidence interval; HbA1C = glycated haemoglobin; NGT=normal glucose tolerance; DM=diabetes mellitus

**Additional file 1: TableS8. Adjusted HR for MACE during 6-year follow-up according to baseline HbA1c level**

**as a continuous variable (log2 transformed)**

|  | Event/Total (%) | HR (95% CI) | P value |
| --- | --- | --- | --- |
| Model 1 | 197/1532 (12.86) | 2.374 (1.329, 4.240) | 0.0035 |
| Model 2 | 197/1532 (12.86) | 2.383 (1.300, 4.366) | 0.0050 |
| Model 3 | 197/1532 (12.86) | 2.150 (1.124, 4.115) | 0.0208 |

The level of fasting glucose is log2 transformed.

Model 1 is univariate analysis;

Model 2 adjusted for age and sex;

Model 3 adjusted for model 2 plus medical history of hypertension, hyperlipidemia, smoking status, alcoholic consumption, body mass index, heart rate, total cholesterol, LDL, HDL, hsCRP, D-Dimer and triple vessel disease;

**Additional file 1: TableS9. Adjusted HR for MACE during 6-year follow-up according to baseline glucose level**

**as a continuous variable**

|  | Event/Total (%) | HR (95% CI) | P value |
| --- | --- | --- | --- |
| Model 1 | 197/1532 (12.86) | 1.031 (0.968, 1.099) | 0.3398 |
| Model 2 | 197/1532 (12.86) | 1.034 (0.969, 1.102) | 0.3119 |
| Model 3 | 197/1532 (12.86) | 1.021 (0.952, 1.094) | 0.5592 |

Model 1 is univariate analysis;

Model 2 adjusted for age and sex;

Model 3 adjusted for model 2 plus medical history of hypertension, hyperlipidemia, smoking status, alcoholic consumption, body mass index, heart rate, total cholesterol, LDL, HDL, hsCRP, D-Dimer and triple vessel disease;

**Additional file 1: TableS10. Subgroup analysis of the association between categories of abnormal glucose metabolism and MACE**

|  | Event/Total (%) | HR (95% CI) | P value | P_interaction_ |
| --- | --- | --- | --- | --- |
| Age ≥ 65 years (N=412) |  |  |  | 0.1867 |
| NGT | 14/98 (14.29) | 1 (reference) | 1 (reference) |  |
| Prediabetes | 22/132 (16.67) | 1.235 (0.614, 2.487) | 0.5539 |  |
| DM | 31/182 (17.03) | 1.429 (0.721, 2.834) | 0.3066 |  |
| Age < 65years (N=1120) |  |  |  |  |
| NGT | 27/429 (6.29) | 1 (reference) |  |  |
| Prediabetes | 36/287 (12.54) | 1.597 (0.959, 2.658) | 0.0718 |  |
| DM | 67/404 (16.58) | 2.058 (1.290, 3.283) | 0.0025 |  |
| Male (N=1044) |  |  |  | 0.2244 |
| NGT | 36/377(9.55) | 1 (reference) | 1 (reference) |  |
| Prediabetes | 47/290(16.21) | 1.597 (0.959, 2.658) | 0.0718 |  |
| DM | 26/127 (20.47) | 2.058 (1.290, 3.283) | 0.0025 |  |
| Female (N= 488) |  |  |  |  |
| NGT | 5/150 (3.33) | 1 (reference) | 1 (reference) |  |
| Prediabetes | 11/129 (8.53) | 2.213 (0.731, 6.696) | 0.1597 |  |
| DM | 31/209 (14.83) | 3.921 (1.438, 10.691) | 0.0076 |  |
| Smoker (N=668 ) |  |  |  | 0.0948 |
| NGT | 19/233(8.15) | 1 (reference) | 1 (reference) |  |
| Prediabetes | 36/170(21.18) | 1.945 (1.100, 3.439) | 0.0221 |  |
| DM | 51/265 (19.25) | 1.827 (1.050, 3.179) | 0.0330 |  |
| Nonsmoker (N=864) |  |  |  |  |
| NGT | 22/294 (7.48) | 1 (reference) | 1 (reference) |  |
| Prediabetes | 22/249 (8.84) | 1.016 (0.554, 1.864) | 0.9590 |  |
| DM | 47/321 (14.64) | 1.981 (1.149, 3.415) | 0.0139 |  |
| Hypertension (N=1013) |  |  |  | 0.2569 |
| NGT | 25/304 (8.22) | 1 (reference) | 1 (reference) |  |
| Prediabetes | 43/272 (15.81) | 1.597 (0.966, 2.642) | 0.0682 |  |
| DM | 72/437 (16.48) | 1.626 (1.012, 2.613) | 0.0445 |  |
| Non-Hypertension (N=519) |  |  |  |  |
| NGT | 16/223(7.17) | 1 (reference) | 1 (reference) |  |
| Prediabetes | 15/147(10.20) | 1.291 (0.615, 2.712) | 0.4991 |  |
| DM | 26/149 (17.45) | 3.009 (1.558, 5.813) | 0.0010 |  |

Adjusted for age, sex, medical history of hypertension, hyperlipidemia, smoking status, alcoholic consumption, body mass index, heart rate, total cholesterol, LDL, HDL, hsCRP, D-Dimer and triple vessel disease;

MACE = major adverse cardiovascular events; HR = hazard ratio; CI = confidence interval; NGT=normal glucose tolerance; NDM=newly-diagnosed diabetes; KDM=known diabetes;


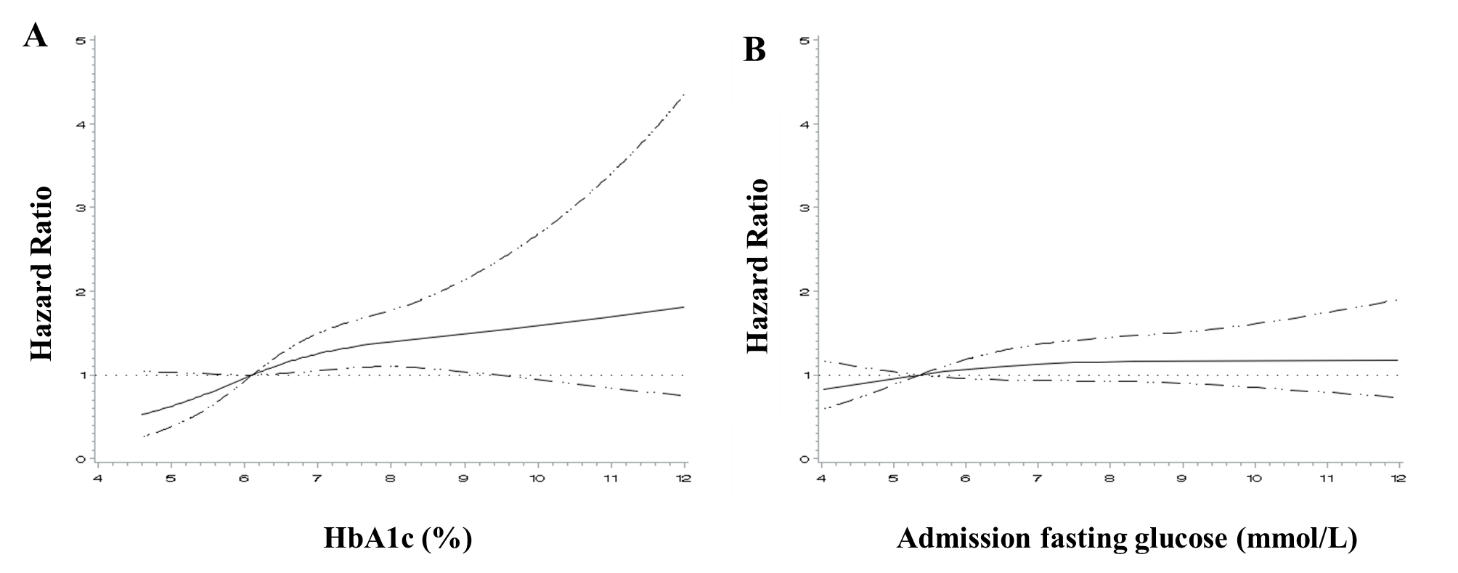


**Additional file 1: FigureS1. Restricted cubic spline analysis of the association between baseline HbA1c level (A) and admission fasting glucose (B) and major cardiovascular event (MACE) risk.** Baseline HbA1c and admission fasting glucose level presented a linear relationship with the risk of MACE (p for non-linearity 0.2119 and 0.4014 respectively). The curves are presented with 95% confidence interval.


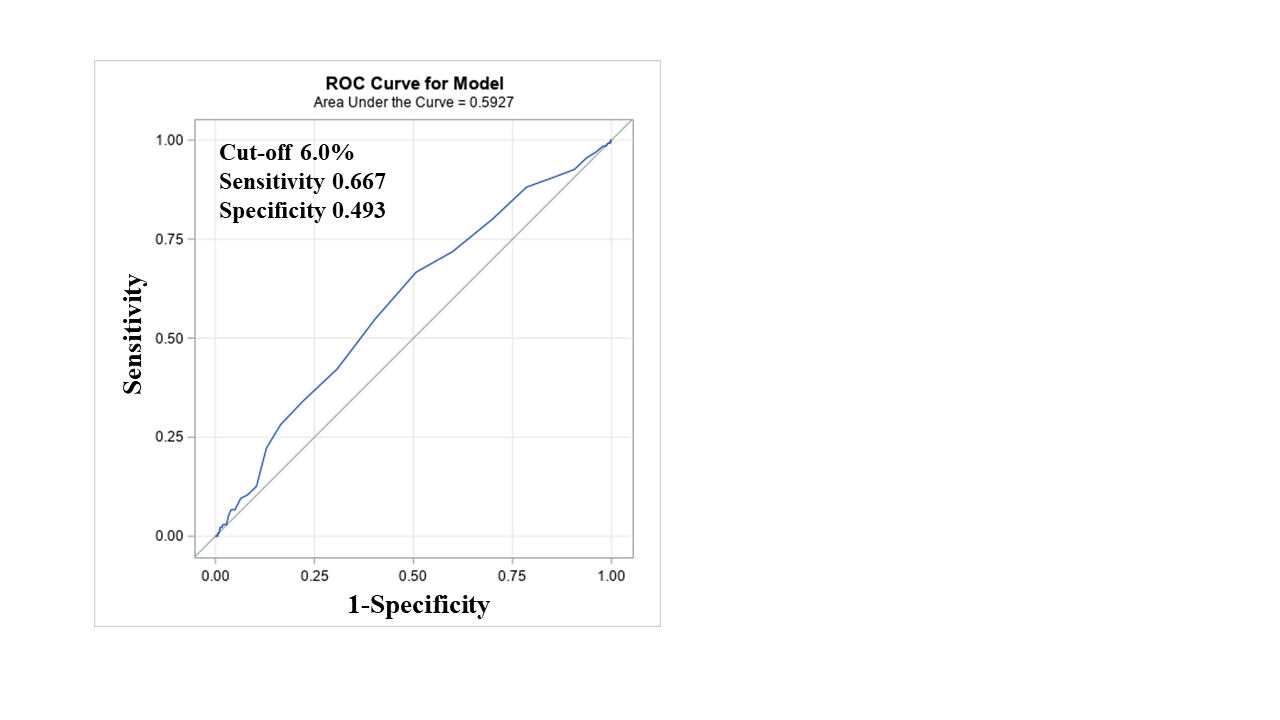


**Additional file 1: FigureS2. ROC Curve of HbA1c in Predicting MACE.** The c-index on the basis of the AUC for HbA1c in predicting ischemic stroke was 0.5927. The best cutoff value of HbA1c based on the highest Youden’s index was 6% with sensitivity of 0.667 and specificity of 0.493.


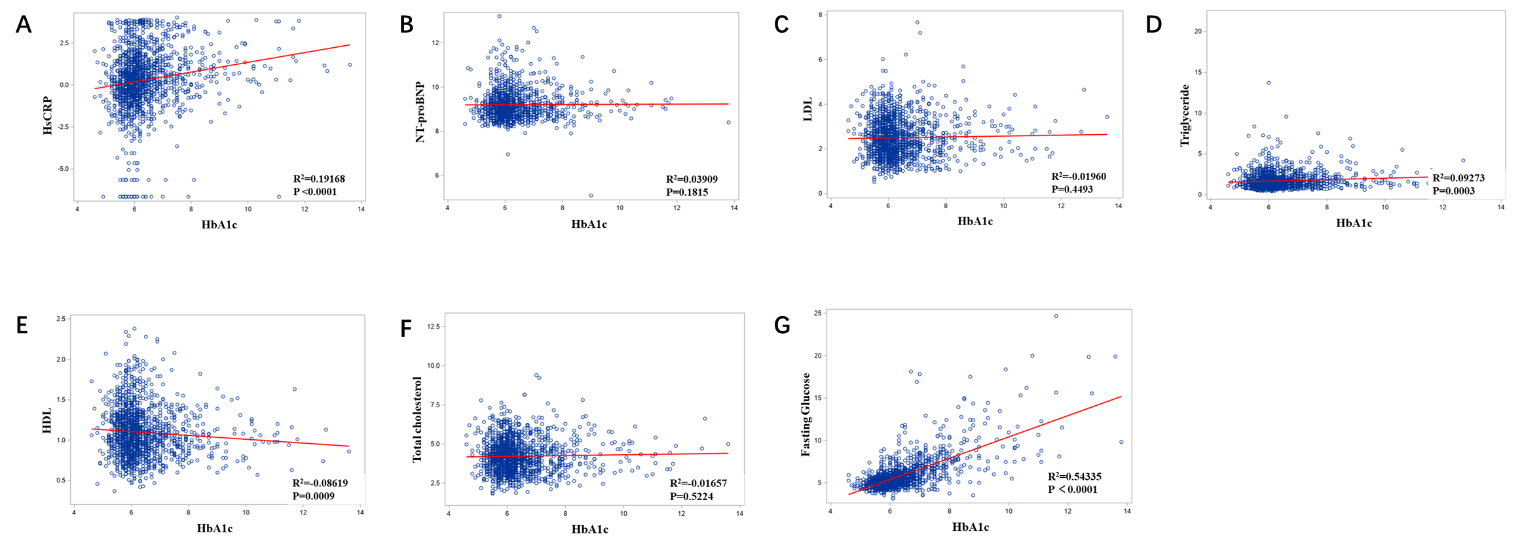


**Additional file 1: FigureS3.** Correlation analysis of the relationship between HbA1c and hsCRP (A), NT-proBNP (B), LDL (C), triglyceride (D), total cholesterol (E), HDL (F) and fasting glucose (G).
